# Supplementary material for: CXCR1 Expression in MDA-PCa-2b Cell Upregulates ITM2A to Inhibit Tumor Growth
Source: Cancers (Basel). 2024 Dec 11;16(24):4138. doi: 10.3390/cancers16244138 (PMC11674668; doi:10.3390/cancers16244138)
Supplement: Supplementary file 1 [file cancers-16-04138-s001.zip › Supplementary Figures S1, S2 & Table S1.pdf]

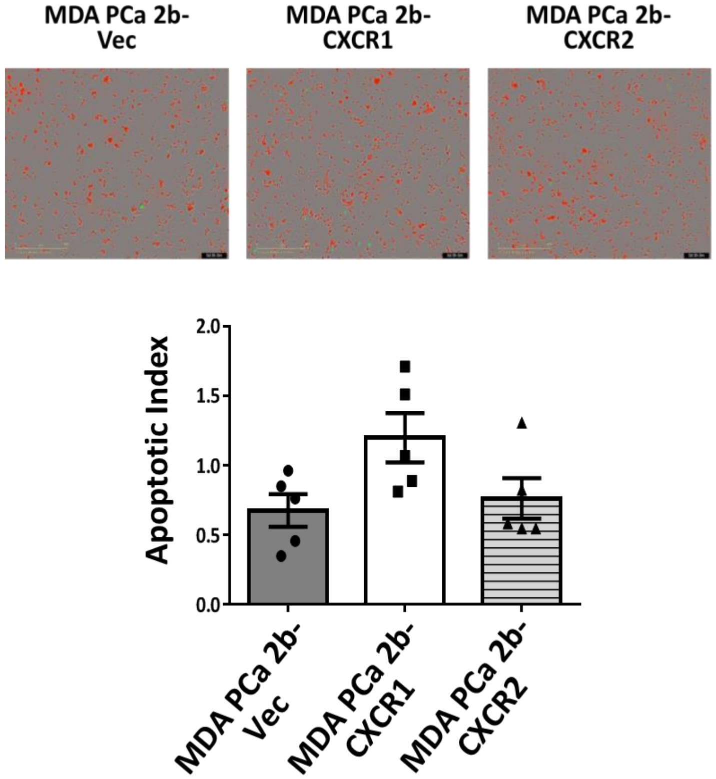

Figure S1. Effect of CXCR1 and CXCR2 overexpression on MDA-PCa-2b cell apoptosis. Representative image and graphical quantification of caspase 3/7 activity measured after staining cells for 30 minutes with CellEvent™ caspase-3/7 green probe and detecting images using the IncuCyte ZOOM® live-cell system. Data are presented as apoptotic index and are representative of two independent experiments. (Green = apoptotic cells; Red = live cells).

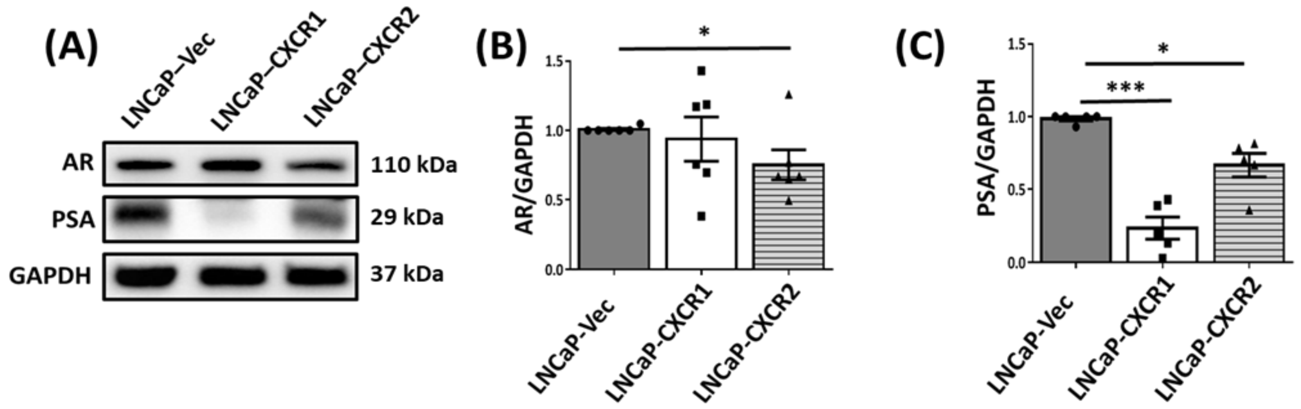

Figure S2. Effects of CXCR1 and CXCR2 overexpression on AR and PSA expression in LNCaP cells A) Cell lysates from LNCaP-Vec, LNCaP-CXCR1 and LNCaP-CXCR2 were assayed for AR, PSA and GAPDH expression by western blotting. Graphical quantification of band density analysis of AR **(B)** and PSA **(C)**, relative to GAPDH are shown. Data shown are representative of at least three different experiments. *$p < 0.05$, **$p < 0.01$, *** $p < 0.001$.

Table S1: Mouse chemokines expression in MDA-PCa-2b-CXCR1 xenografts relative to control MDA-PCa-2b-Vec cells

Chemokine	Fold Change over Control
CCL21	224
CXCL13	66
CCL6	17
Chemerin	5
CCL27	2.4
CXCL16	6.3
CCL11	18
IL-16	10.4
CCL8	2.8
CCL12	4
CXCL9	2
CCL9/10	9.34
CCL5	32
CXCL12	6
Factor D	2.88
