# Supplementary material for: CXCR1 Expression in MDA-PCa-2b Cell Upregulates ITM2A to Inhibit Tumor Growth
Source: Cancers (Basel). 2024 Dec 11;16(24):4138. doi: 10.3390/cancers16244138 (PMC11674668; doi:10.3390/cancers16244138)
Supplement: Supplementary file 1 [file cancers-16-04138-s001.zip › Supplementary Figures S3 - S11.pdf]

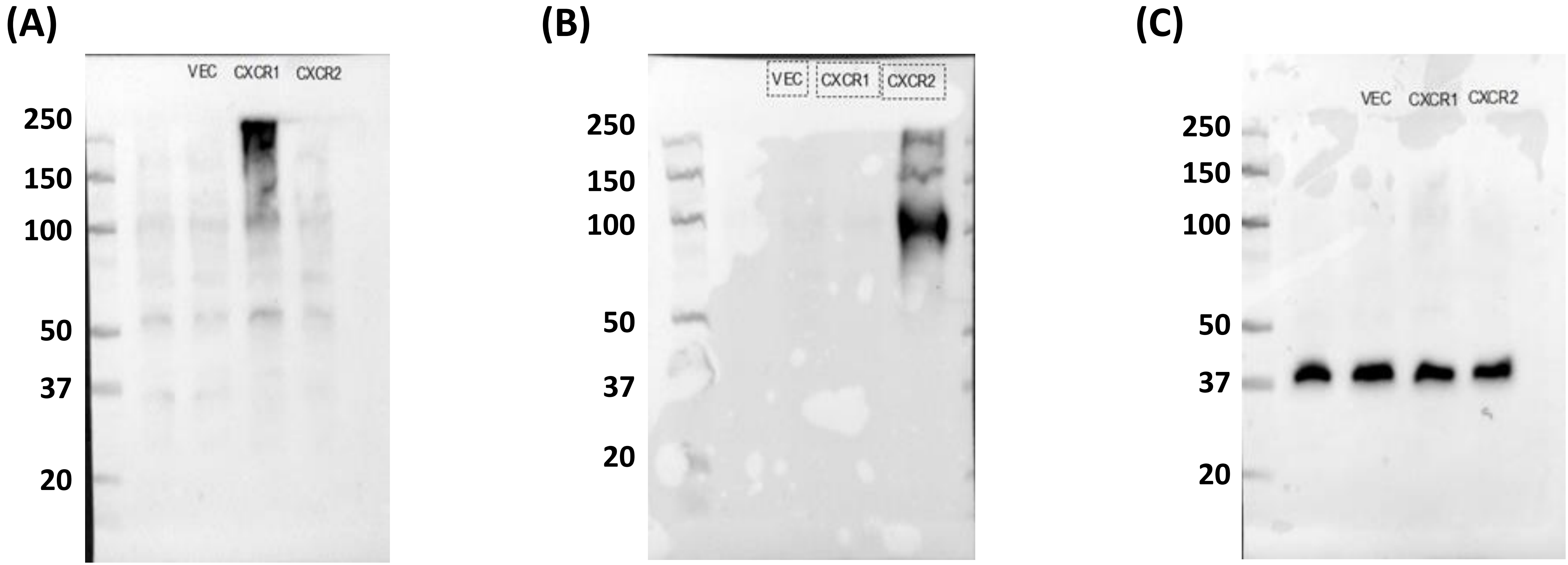

Figure S3: The original Western Blotting Images of CXCR1 (A), CXCR2 (B) and GAPDH (C) for Figure 1B

(A) (B)

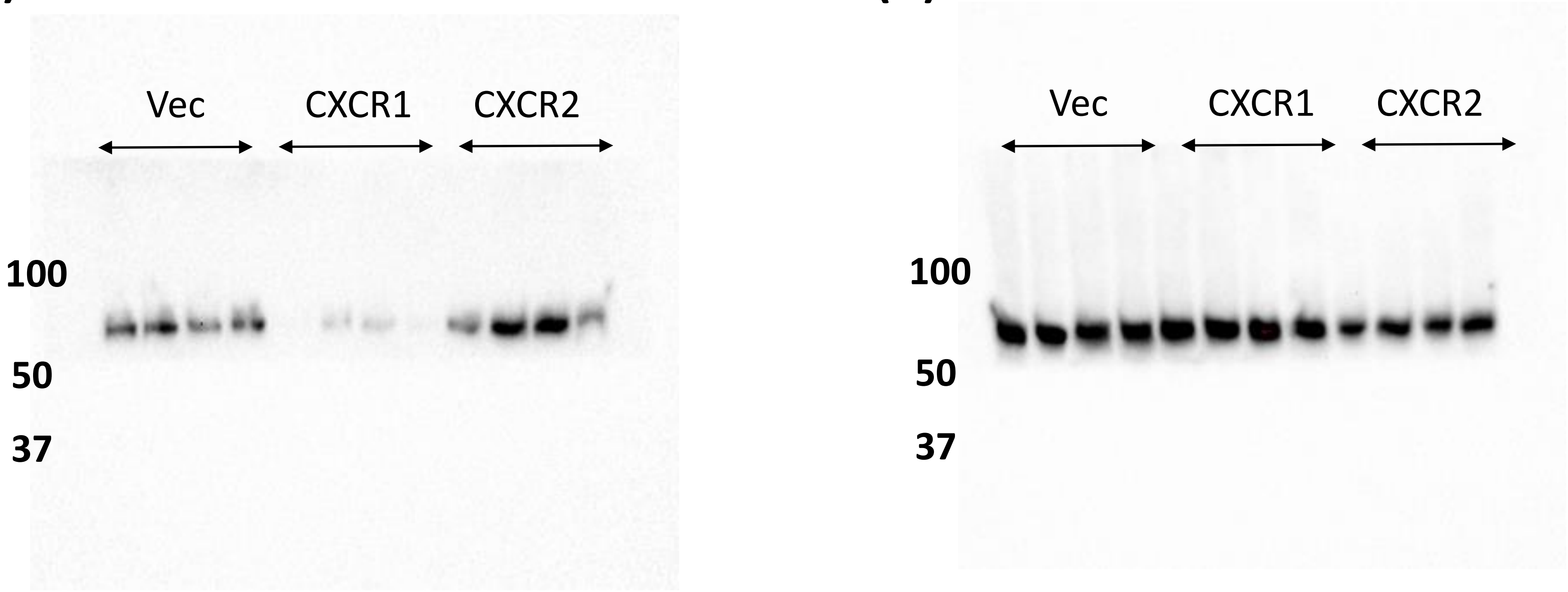

Figure S4: The original Western Blotting Images of p-AKT (A) and Total AKT (B) proteins for Figure 1E

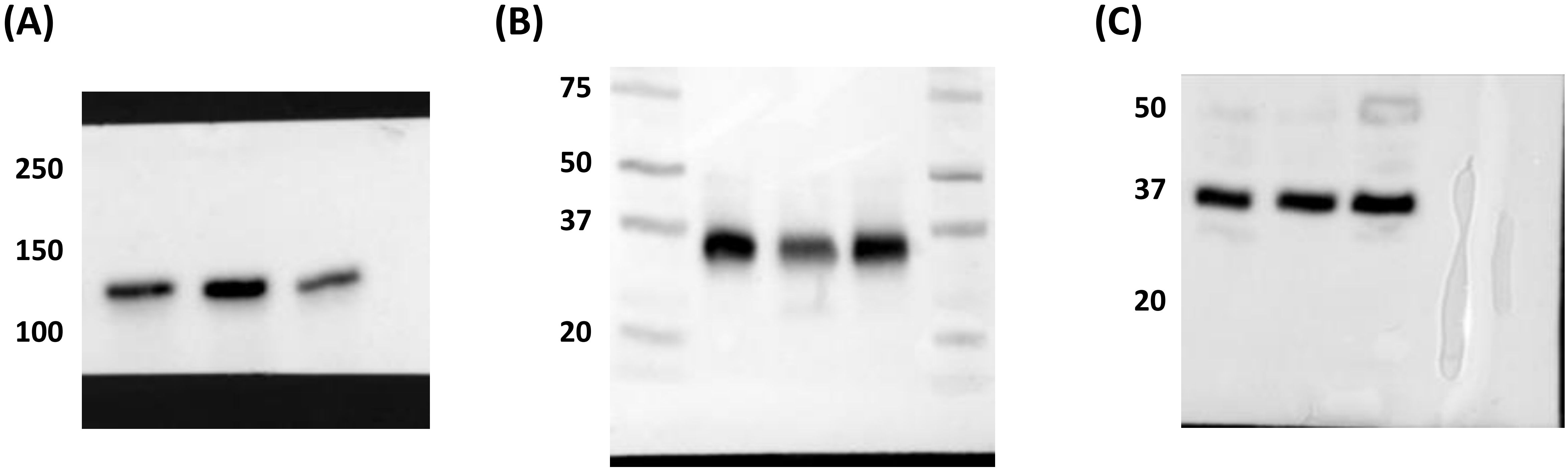

Figure S5: The original Western Blotting Images of AR (A), PSA (B) and GAPDH (C) for Figure 2A

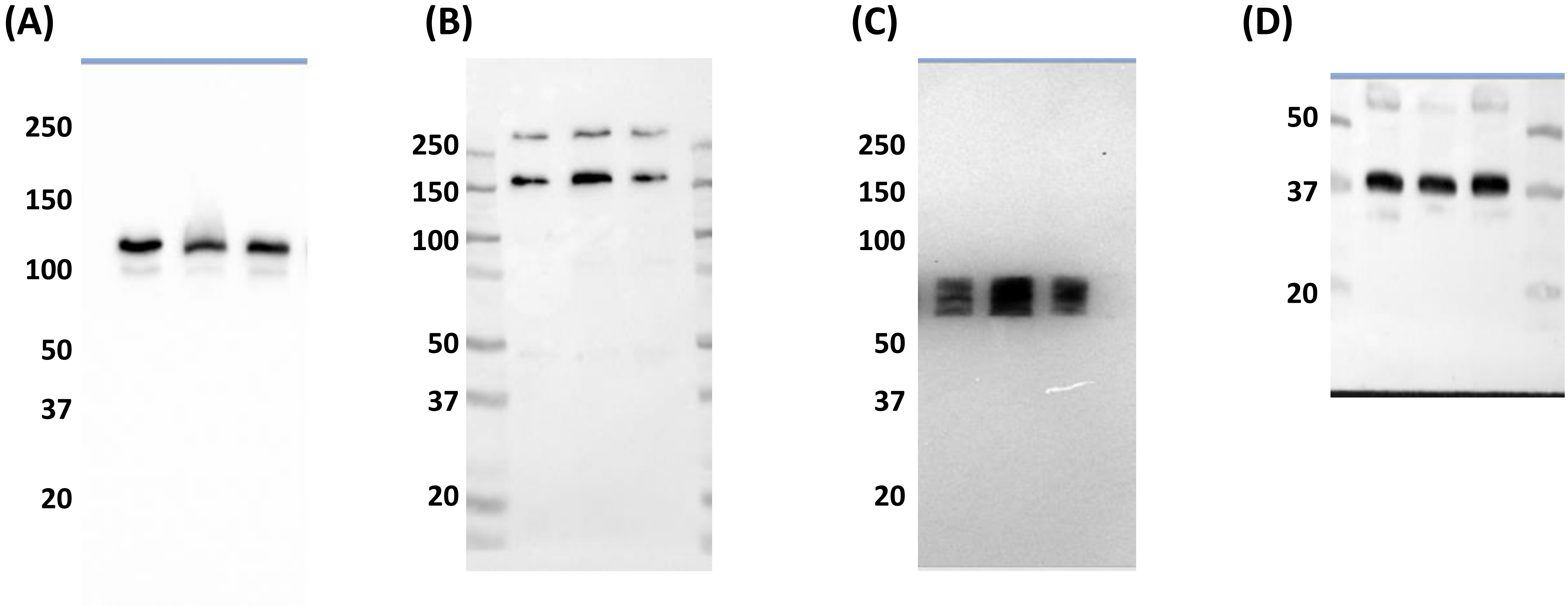

Figure S6: The original Western Blotting Images of E-Cadherin (A), N-Cadherin (B), Vimentin (C) and GAPDH (D) for Figure 2D

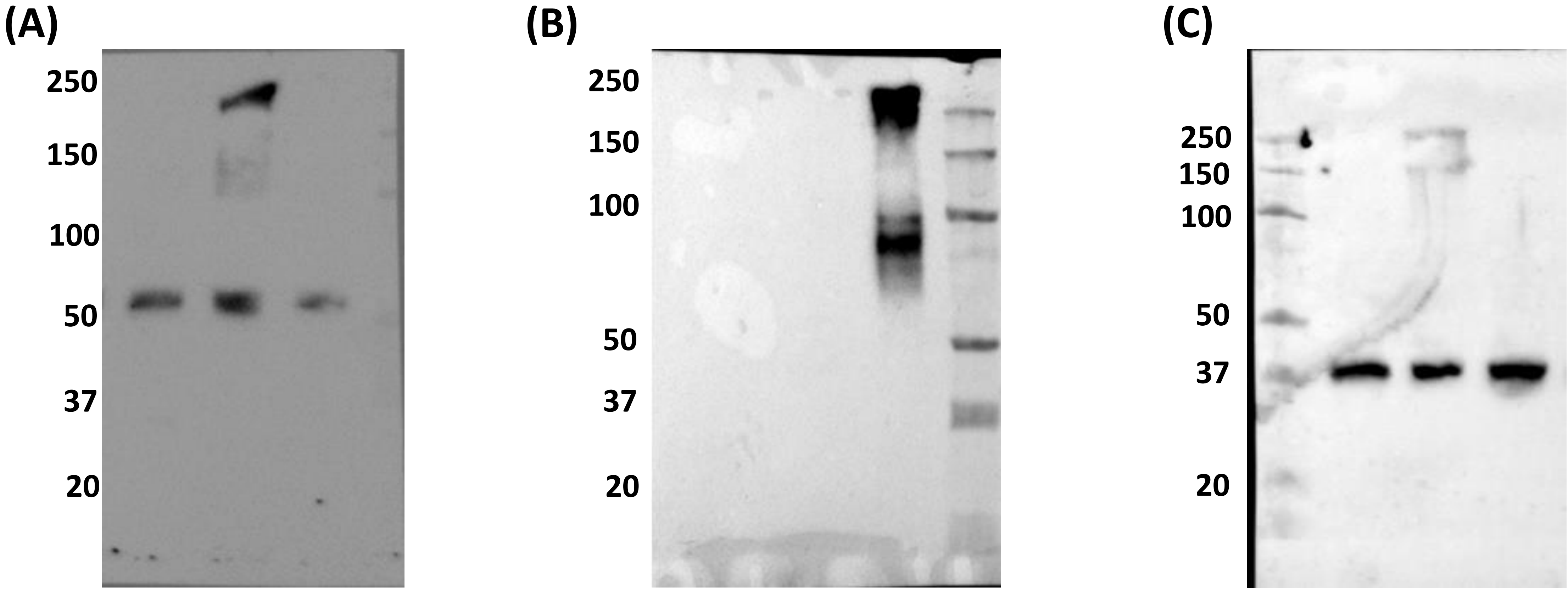

Figure S7: The original Western Blotting Images of CXCR1 (A), CXCR2 (B) and GAPDH (C) for Figure 4B

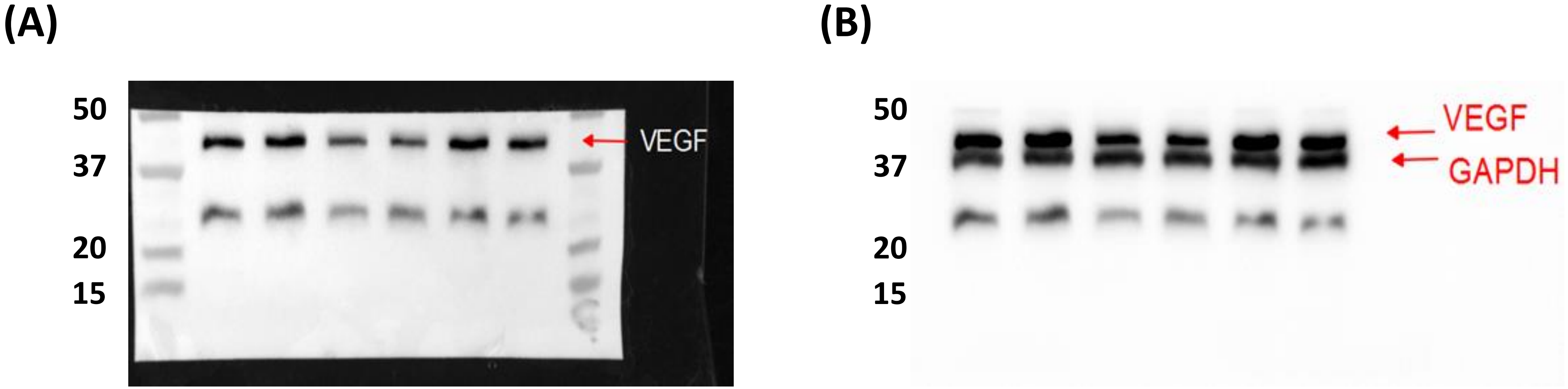

Figure S8: The original Western Blotting Images of VEGF (A), and GAPDH (D) for Figure 5F

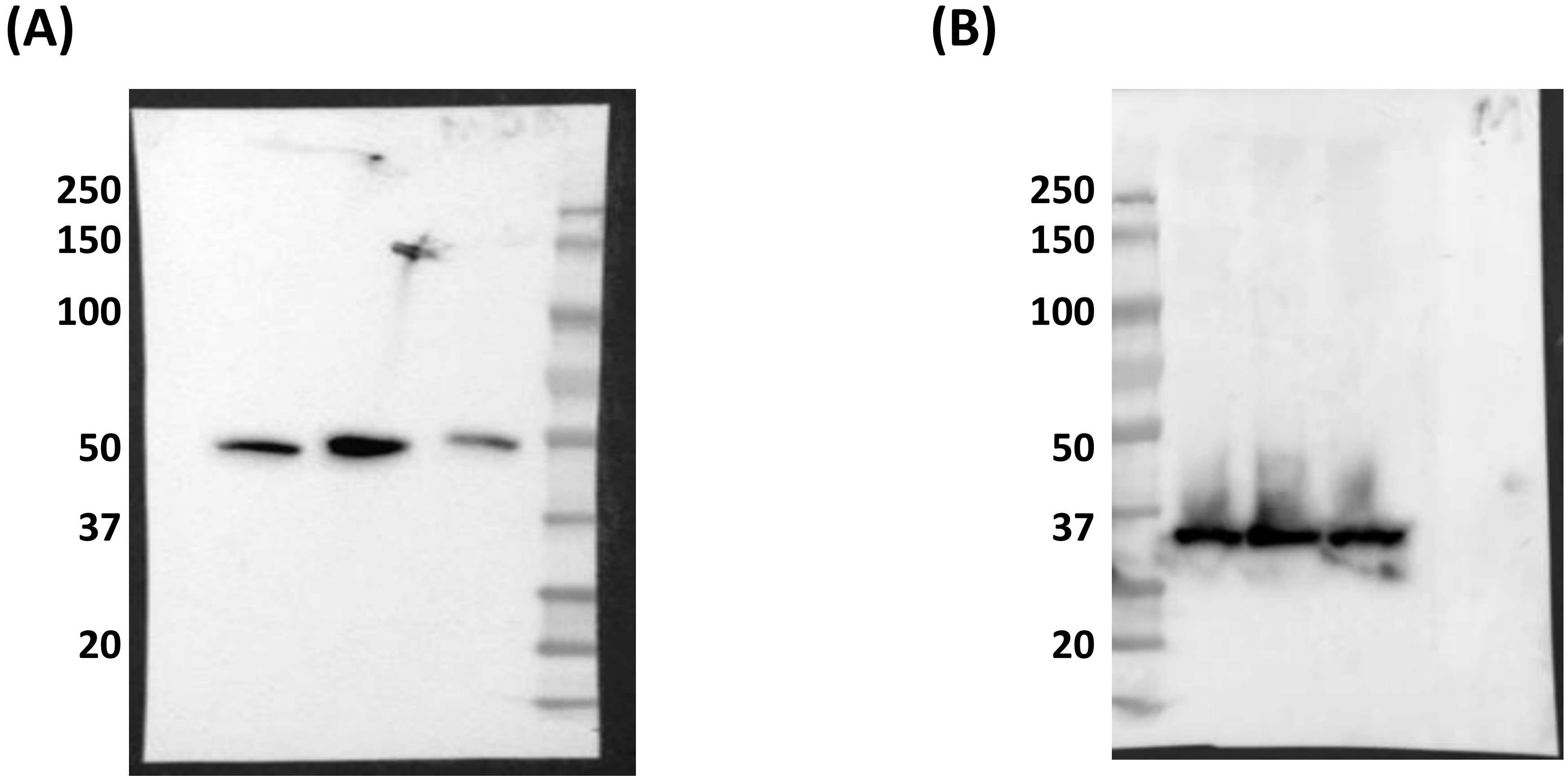

Figure S9: The original Western Blotting Images of ITM2A (A), and GAPDH (D) for Figure 6E

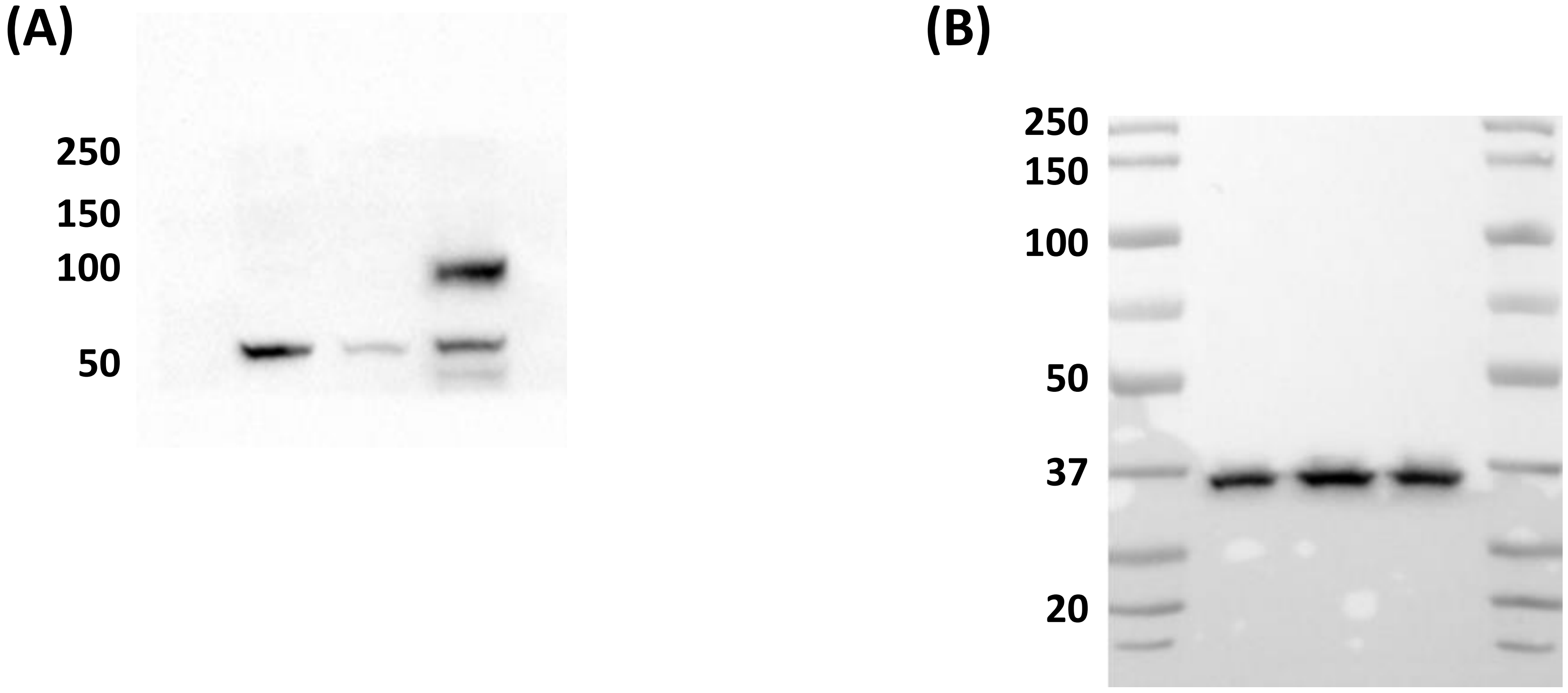

Figure S10: The original Western Blotting Images of ITM2A (A), and GAPDH (D) for Figure 7A

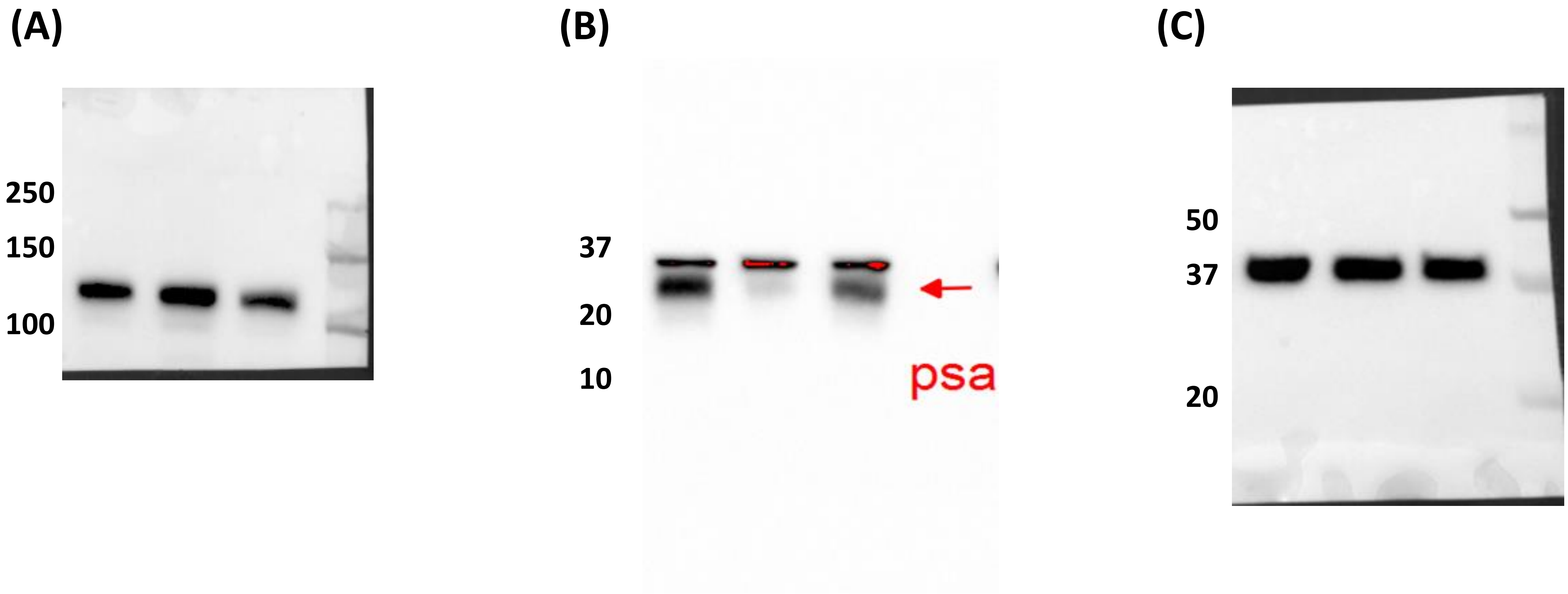

Figure S11: The original Western Blotting Images of AR (A), PSA (B) and GAPDH (C) for Figure S2A
