# Supplementary figures and images for: CXCR1 Expression in MDA-PCa-2b Cell Upregulates ITM2A to Inhibit Tumor Growth
Source: Cancers (Basel). 2024 Dec 11;16(24):4138. doi: 10.3390/cancers16244138 (PMC11674668; doi:10.3390/cancers16244138)

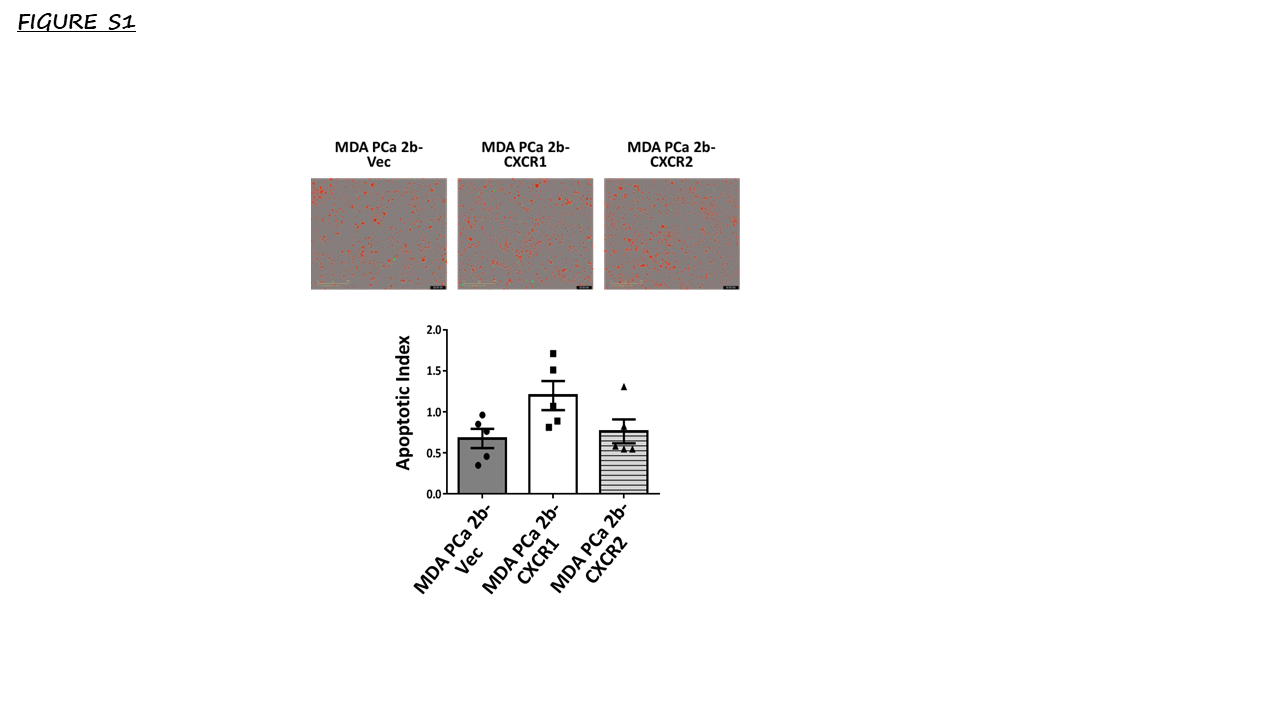

Supplement: Supplementary file 1 [file cancers-16-04138-s001.zip › Figure S1.TIF]

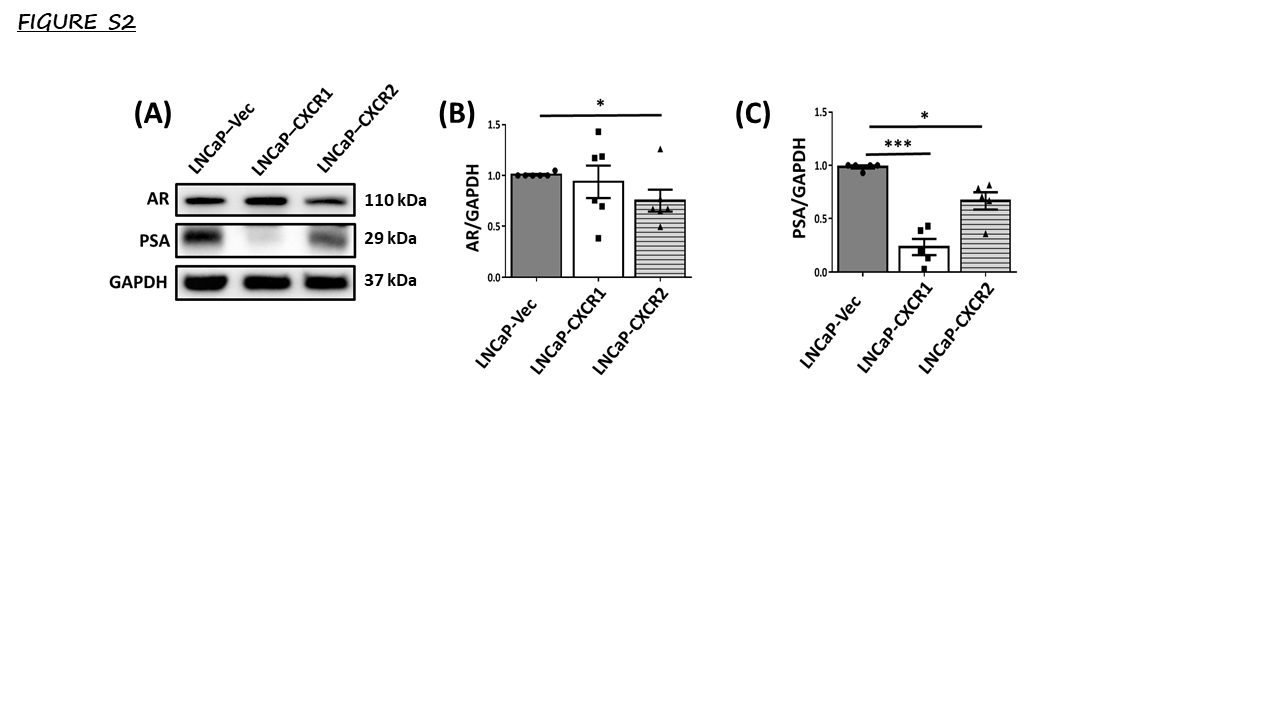

Supplement: Supplementary file 1 [file cancers-16-04138-s001.zip › Figure S2.TIF]
